#  The Neuroprotective Effect of Rosemary (*Rosmarinus officinalis *L.) Hydro-alcoholic Extract on Cerebral Ischemic Tolerance in Experimental Stroke

**Published:** 2016

**Authors:** Parisa Seyedemadi, Mehdi Rahnema, Mohammad Reza Bigdeli, Shahrebano Oryan, Hassan Rafati

**Affiliations:** a *Department of Physiology, Faculty of Biological Sciences, Kharazmi University, Tehran, Iran. *; b *Department of Physiology, Faculty of Biological Sciences, Shahid Beheshti University, G.C., Tehran, Iran. *; c *Department of Physiology, Islamic Azad University – Zanjan Branch, Zanjan, Iran. *; d *Department of Chemical Engineering, Medicinal Plants & Drugs Research Institute, Shahid Beheshti University, Tehran, Iran.*

**Keywords:** Rosemary leaf hydro-alcoholic extract, Cerebral ischemia reperfusion, Infarct volume, Brain edema, Blood–brain barrier permeability, Neuro-protective

## Abstract

The prevention of BBB breakdown and the subsequent vasogenic edema are important parts of the medical management of ischemic stroke. The purpose of this study was to investigate the ischemic tolerance effect of *Rosmarinus officinalis* leaf hydro-alcoholic extract (RHE).

Five groups of animals were designed: sham (underwent surgery without MCAO) and MCAO groups, the MCAO groups were pretreated orally by gavages with RHE (50, 75, and 100 mg/Kg/day), daily for 30 days. Two hours after the last dose, serum lipid levels were determined and then the rats were subjected to 60 min of middle cerebral artery occlusion followed by 24 h of reperfusion. Subsequently, brain infarct size, brain edema and Evans Blue dye extravasations were measured and neurological deficits were scored.

Dietary RHE could significantly reduce cortical and sub-cortical infarct volumes (211.55 ± 24.88 mm^3^ *vs*. 40.59 ± 10.04 mm^3 ^*vs*. 29.96 ± 12.19 mm^3^*vs*. 6.58 ± 3.2 mm^3^), neurologic deﬁcit scores, cerebral edema (82.34 ± 0.42% *vs*. 79.92 ± 0.49% *vs*. 79.45 ± 0.26% vs. 79.30 ± 0.19%), blood–brain barrier (BBB) permeability (7.73 ± 0.4 μg/g tissue *vs*. 4.1 ± 0.23 μg/g tissue *vs*. 3.58 ± 0.3 μg/g tissue *vs*. 3.38 ± 0.25 μg/g tissue) in doses of 50, 75 and 100 mg/Kg/day as compared with the control group in the transient model of focal cerebral ischemia.

Although pretreatment with RHE plays an important role in the generation of tolerance against cerebral I/R injury, further studies are needed to clarify the mechanism of the ischemic tolerance.

## Introduction

Acute ischemic stroke is a major cause of both death and disability (1). Nearly 85% of strokes are acute ischemic strokes which result from a sudden loss of blood supply to the cerebral regions (2). This, deprives the brain of oxygen and nutrients and initiates a dynamic sequence of patho-physiological events (3). Oxidative stress and inflammatory events play an integral role in the pathogenesis of cerebral ischemia (4).

Disruption of blood–brain barrier (BBB), subsequent edema is major contributors to the pathogenesis of ischemic stroke, the details for pathogenesis of ischemic stroke remains unelucidated (5). Edema formation after ischemia can cause an increase in brain mass (1), which in turn leads to cell death and deterioration of stroke (6). Thus, protection of BBB breakdown by natural products may be a promotional strategy for treatment of ischemic tolerance (5).

The current strategies of therapeutic management such as tPA have the potential to aggravate ischemic brain injuries by increasing infarct size, cerebral edema, and intracranial hemorrhage (7). Normally, the infarct core will grow and progressively replace the penumbra (8). Therefore, rescuing the ischemic penumbra from infarction by potentiating of ischemic tolerance in this tissue is the main goal of ischemic tolerance (9).

There is a signiﬁcant increase in the research and development of herbal medicine based on scientific studies as a supplement (10). Rosemary (*Rosmarinus ofﬁcinalis* L.) is an important medicinal herb from Lamiaceae family originated from Mediterranean region and has been cultivated for long time in Iran (11). 

Phytochemical studies have shown that rosemary (*Rosmarinus officinalis* L.) extracts (REs) contain essential oils, terpenoids, ﬂavonoids, and alkaloids (12), including carnosic acid (CA), rosmarinic acid, carnosol (CS), caffeic acid, and ursolic acid, which contribute to the biological activity of rosemary and could provide effective synergy (13).

It is well known that the aqueous and alcoholic extracts of the leaves of R. *ofﬁcinalis* L. possess a variety of pharmacological properties, including anti-aging (14), hepatoprotective, anti-bacterial, anti-thrombotic, anti-ulceogenic, diuretic, anti-diabetic, antioxidant, anti-noceptive, anti-inﬂammatory, and anti-depressant activities (15). On the other hand, the bioactive components of REs exhibit potent antioxidant activities (16), reduce lipid peroxidation in heart and brain (cortex and hippocampus) (17), inhibit the production of reactive oxygen species, and suppress inﬂammatory response (18). 

It has been reported that REs can be useful in the prevention of disorders due to angiogenesis (19) and atherosclerosis (20). 

The effects of *Rosmarinus officinalis* leaf hydro-alcoholic extract (RHE) on ischemic–reperfusion injury of the rat brain still remain to be explored. We hypothesized whether RHE can induce ischemic tolerance for protecting the brain from focal ischemic stroke. Therefore, the aim of this study was to examine the effects of various doses of hydro-alcoholic extract from rosemary (*Rosmarinus officinalis* L.) leaf on BBB permeability, brain edema, brain injuries and neurological dysfunctions during the acute phase of injury following focal cerebral ischemia reperfusion in rats.

## Materials and methods

*Preparation of extract*

The Arial parts of the cultivated *Rosmarinus officinalis* were collected in August 2011 from the Herb Farm of Shahid Beheshti University. Voucher specimen of the collected plants (voucher no; 1922 ) was confirmed at Medicinal Plant Herbarium (MPH), Department of Phytochemistry, Medicinal Plants and Drugs Research Institute, Shahid Beheshti University.

Dried and powdered rosemary leaves (500 g) were extracted using enough volume of ethanol/water (70:30) kept at dark room with temperature (23 °C) for 48 h. The resulting solution was filtered through filter paper. After filtration, the solvent was removed by rotary evaporator to produce an waxy semi- solid materials. The rosemary extract was dissolved in 0.2% Tween 80 and distilled water added as vehicle for oral (p.o.) grouping. 

*High performance liquid chromatography (HPLC)*

A reverse phase HPLC analysis (RP-HPLC) was used to quantify the major active components of the extract. The HPLC system consisted of a K-1001 pump, a K-2800 photodiode array (PDA) detector Knauer (Berlin, Germany). The chromatographic separations were performed on Eurosphere C18 (5 µM, 250 mm × 4.6 mm), Knauer (Berlin, Germany). Data were acquired and processed using a Chromgate software. The mobile phase composed of 0.01% formic acid solution and methanol from 75:25 to 20:80 %v/v gradient over 30 min at a flow rate of 0.5 mL min^−1^ and column temperature of 25 °C. Detector response was set at 330 nm. RHE contains 1.3% caffeic acid (Sigma, London, UK), and 4% rosmarinic acid (Merck, Darmstadt, Germany). 

*Animals*

All the experimental and animal handling procedures were approved by the Animal and Ethics Review Committee of the University of Kharazmi of Iran. Adult male Wistar rats (Razi Institute, Tehran, Iran) weighing 280-300 g were housed in standard cages in a temperature (22 ± 2 °C) with humidity (40 ± 5%), under a 12 h light/dark cycle (lights on at 07:00), and received standard diet and water freely. 

## Experimental

Animals were randomly divided into four groups (n = 18 in each group).The first group served as control and vehicle was given orally (p.o.); the other three groups were pretreated orally (p.o.) once daily (10-11.00 h), for 30 days with RHE (50, 75 and 100 mg/Kg body weight): doses identified using previously published data. 2 h after the last pretreatment, the rats subjected to 60 min of middle cerebral artery occlusion followed by 24 h of reperfusion. After 24 h reperfusion, neuro-behavioral studies, infarct volume (n = 6), water content (n = 6), and blood brain barrier permeability (n = 6) were measured. Additionally, 12 sham-operated animals underwent the same surgical procedures (without blocking the arteries).

*Lipid proﬁles*

The rats were anesthetized with chloral hydrate (400 mg/Kg i.p.) at 30 days after the initiation of pretreatment, just before surgery. Then, blood samples were taken by tail vein. The blood samples were centrifuged at 7000 × g for 10 min, room temperature to obtain plasma. 

The serum samples were stored at -20 °C until biochemical analysis. Levels of HDL, triglyceride, and cholesterol content (Pars Azmun, Iran according to manufacturer’s instructions) were measured using an autoanalyzer (Liasys, Roma, Italy). 

*Middle cerebral artery occlusion*

The right middle cerebral artery (MCA) occlusion (MCAO) was induced using an intraluminal ﬁlament model (21). The rats were anesthetized with chloral hydrate (Merck, Germany; 400 mg/kg i.p.). Briefly stating, neck vessels were exposed through a midline incision. Under an operating microscope, the right common carotid artery (CCA), the right external carotid artery (ECA), and the right internal carotid artery (ICA) were isolated. Then, a 3-0 silicone-coated nylon was carefully inserted from the external carotid artery into the internal carotid artery (ICA) until light resistance was felt. The ﬁlament was inserted approximately 20 mm from the carotid bifurcation to effectively block the middle cerebral artery (MCA). After 60 min of transient MCA occlusion (tMCAO), blood flow was restored by withdrawing the nylon filament and the ECA was permanently tied. During these procedures, body temperature was monitored with a rectal probe, and was maintained at 37±0.3 °C (Citizen-513w) using a heating pad. Animals were then recovered from anesthesia and were returned to their cages for 24 h. Sham animals were subjected to surgery with sutures of the same size but the suture was not advanced into the middle cerebral artery. The animals were kept at ambient temperature until sampling, with free access to water and food.

*Neurological examination*

Neurological function was evaluated using a 0-5 point scale neurological score (21) after 24 h of reperfusion: 0 = no neurological dysfunction; 1 = failure to extend opposite forepaw; 2 = circling to the contralateral side, when held by tail with feet on ﬂoor; 3 = falling to the left; 4 = unable to bear weight on affected side; 4 = no spontaneous walking and a depressed level of consciousness; 5 = death. 

*Measurement of infarct volume*

The rats were decapitated under chloral hydrate anesthesia (800 mg/Kg), 24 h post-stroke and the brains were rapidly removed. The brains were cooled in ice-cold saline for 10 min and sectioned coronally into eight 2 mm-thick slices starting from the forebrain area (i.e. from the olfactory bulbs to the cortical-cerebellar junction),by using a Brain Matrix Slicer. Brain slices were incubated for 20 min in a 2% solution of 2, 3, 5-triphenyl tetrazolium chloride (Merk, Germany) and kept at 37 °C in a water bath for 15 min. The stained brains were then photographed using a digital camera connected to a computer (Cannon, Japan). Damaged regions were deﬁned as areas that were completely white. The infarct area in each section was measured using image analysis software (UTHSCSA Image Tool). The infarct volume was calculated by taking the average of infarct area on both sides of the slice and multiplying it by section thickness. The total infarct volumes of each brain were calculated as the sum of the infarct volumes of each slice (22): corrected infarct volume = left hemisphere volume-(right hemisphere volume - infarct volume).

*Water content of the brain*

The rats were decapitated at 25 h after reperfusion and the brains were removed. Then, the pons and olfactory bulb were removed and the brain tissues were weighted immediately to obtain their wet weight (WW) (23). Subsequently, brains were dried at 110 °C for 24 h in an oven to determine their dry weight (DW). Brain water content (BWC), was calculated according to the formula: (WW−DW)/WW×100.

*Evaluation of BBB permeability disruption*

Measurement of Evans blue extravasations BBB permeability was assessed by measuring EB extravasations. Briefly stating, 4 mL/Kg of 2% Evans blue solution (EB, Sigma Chemicals, USA) in saline was injected into the tail vein, 30 min before reperfusion. Twenty-four h after, rats were deeply anesthetized, chest walls were opened, and trans-cardially perfused with 250 mL saline solution. After decapitation, the brain tissue was removed and hemispheres were separated and weighed. Each hemisphere was homogenized, in the 2.5 mL phosphate buffer saline, and then 2.5 mL of 60% trichloroacetic acid (Merck, Germany) was added to precipitate protein. The mixture was then vortexed for 3 min, cooled for 30 min and centrifuged for 30 min at 1000 ×g. The supernatants were measured at 610 nm for absorbance of EB using a spectrophotometer (UV-visible, USA). The results were expressed as microgram per gram brain tissue calculated according a standard curve (23).

*Statistical analysis*

All data are presented as the mean± S.E.M. Statistical analysis of neurological score was assessed using analysis of variance followed by a post hoc Fisher’s indicated least signiﬁcant difference . For neurological score, we have applied Mann-Whitney U test. A *p*-value of less than 0.05 was considered to be statistically significant. 

## Results

*Effects of RHE on body weight*

The pretreatment with RHE at doses of 50, 75 mg/Kg/day for 30 days did not decrease body weight as compared with control and same pretreatment groups (Table 1.), but 100 mg/Kg/day of RHE significantly decreased body weight one day before the end of study (day 30).

*Effects of the RHE pre-treatment on serum lipid levels*

**Figure 1:**
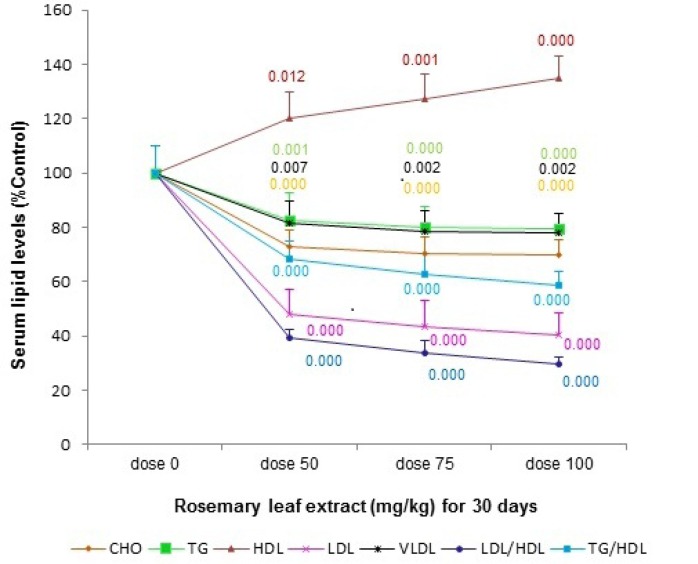
the effect of various doses (0, 50, 75, and 100 mg/Kg) of rosemary leaf hydro-alcoholic extract (RHE) pretreatment for 30 days on serum lipid levels. The data are expressed as the mean ±S.E.M. (*p-values* *are* *colored* *based* *on* *their* *curves*

The total cholesterol (TC), triglyceride (TG), LDL-c levels were significantly reduced in the serum of rats supplemented with RHE at the doses of 50, 75, and 100 mg/Kg/day for 30 days as compared to the vehicle-treated rats. RHE pretreatment increased the level of serum HDL-c when compared with the vehicle-treated group. A significant reduction of the LDL/HDL-c and TG/HDL-c ratios was also seen in all consuming RHE groups as compared to control group at the end of thirtieth day (Figure 1.).

*Effect of RHE on neurologic outcome*

**Figure 2:**
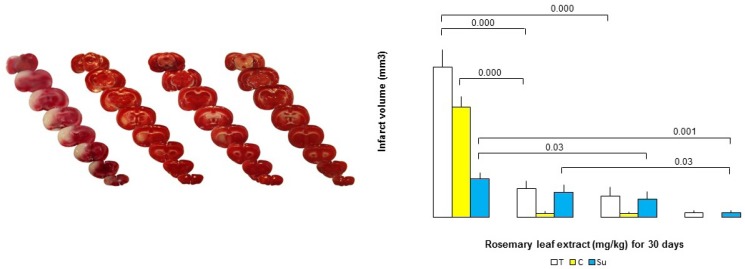
A) Representative TTC-stained brain coronal sections at 24 h after 1 h of transient middle cerebral artery occlusion (tMCAO): (a,b,c, and d) related brain sections of rosemary leaf hydro-alcoholic extract (RHE) (0, 50, 75, and 100 mg/Kg/day) pretreated rats, respectively. White areas show damaged regions; red areas show surviving regions. B) Graph displaying brain infarct volumes in the total hemisphere (T), cortex (C), and sub-cortex (Su) in the MCAO group rats pretreated with RHE at the doses of 50, 75, and 100 mg/ Kg/day, as compared to the corresponding infarct volume of control MCAO group rats (n = 6).

Median neurologic deficit scores (NDS) were reduced by RHE, being 1 (range: 0-2), 1 (range: 0-2), 1 (range: 0-1), and 2.5 (range: 0-4) in the doses of 50, 75 and 100 mg/Kg/day and control (0) for 30 days, respectively (Table 2.). The putative beneficial effects of RHE were confirmed by a reduction in infarct volume (Figure 2.).Therefore, the neurologic deﬁcit scores (NDS) in the MCAO group pretreated with RHE at the doses of 50, 75, and 100 mg/Kg/day, for 30 days were reduced in comparison with the vehicle-pretreated MCAO group.

*Dose-dependent effect of RHE on Infarct volume*

In the control group, 1h-ischemia/24 h-reperfusion caused severe infarct in the cerebral cortex and subcortex areas. RHE pretreatment at doses of 50, 75 and 100 mg/Kg/day for 30 days reduced infarct volumes markedly with maximal protection at the doses of 75 and 100 mg/Kg/day as compared to the controls (Figure 2.). The neuro-protection, exerted by RHE was mainly seen in the cortex, while it was slightly observed in the infarct sub-cortex. 

*Effect of RHE on brain edema at 24 h post-tMCAO*

**Figure 3:**
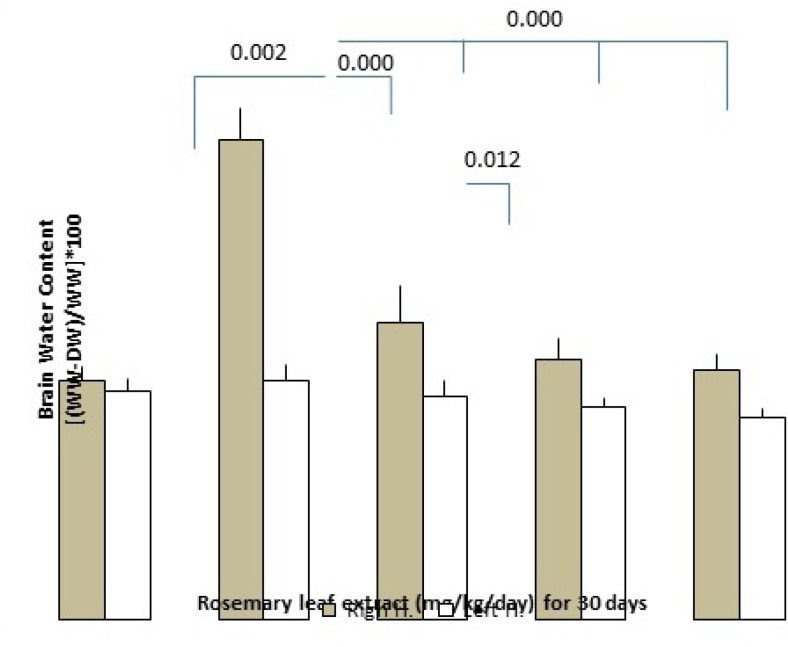
the effect of RHE pretreatment for 30 days on brain water content including left and right hemisphere of the control and RHE at the doses of 50, 75 and 100 mg/Kg/day groups as compared with the sham-operated group (n = 6

Brain edema formation following induction of cerebral ischemia/reperfusion was determined by the measurement of the brain water content. Pretreatment of RHE at the doses of 50, 75, and 100 mg/Kg/day, reduced edema formation significantly as compared with control rats (the dose of 50 mg/Kg/day: left hemisphere = 78.95 ± 0.2%, right hemisphere = 79.92 ± 0.49%; the dose of 75 mg/Kg/day: left hemisphere = 78.81 ± 0.1%, right hemisphere = 79.45 ± 0.26%, dose of 100 mg/Kg/day: left hemisphere =78.68 ± 0.1%, right hemisphere = 79.3 ± 0.19% and in the control group: left hemisphere = 79.82±0.19%, right hemisphere = 82.34 ± 0.42%)(Figure 3.).

*Effect of RHE on BBB dysfunction in stroke*

**Figure 4:**
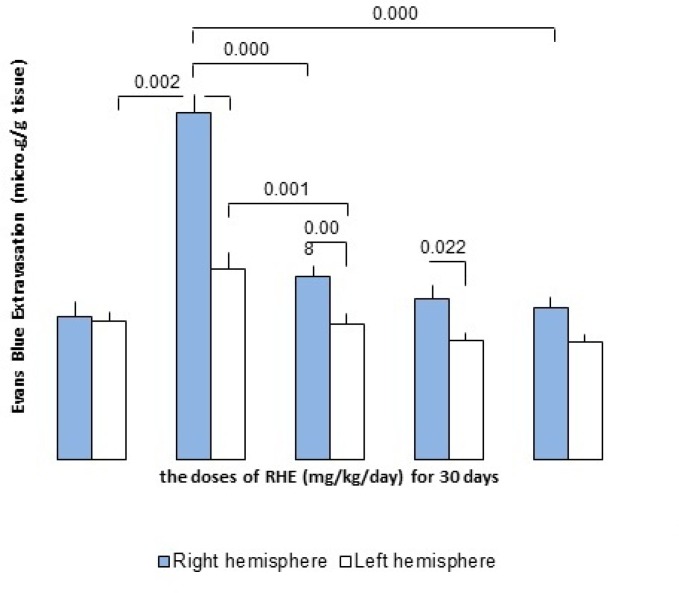
the effect of RHE pretreatment for 30 days on regulation of BBB integrity including left and right hemisphere of control and experimental groups (RHE at the doses of 50, 75 and 100 mg/Kg/day-pretreated MCAO) as compared to the sham-operated group (n = 6).

The permeability of BBB followed by ischemia/reperfusion was evaluated by the amount of Evans Blue (EB) extravasations in the ischemic right hemisphere. In the MCAO-operated (control) group, EB content in the right cerebral hemisphere (ischemic) was 7.73 ± 0.4 and 4.25 ± 0.35 μg/g brain tissues in the left cerebral non-ischemic hemisphere. In contrast, RHE pretreatment at doses of 50, 75 and 100 mg/Kg/day for 30 days was significantly reduced extravasations of EB dye into the ischemic brain (The dose of 50 mg/Kg/day: non-ischemic hemisphere = 3.02 ± 0.25 μg/g tissue, ischemic hemisphere = 4.1 ± 0.23 μg/g tissue; the dose of 75 mg/Kg/day: non-ischemic hemisphere= 2.65 ± 0.18 μg/g tissue, ischemic hemisphere = 3.58 ± 0.3 μg/g tissue and dose of 100 mg/Kg/day: non-ischemic hemisphere = 2.63 ± 0.17 μg/g tissue, ischemic hemisphere= 3.38 ± 0.25 μg/g tissue)(Figure 4.).

## Discussion

In the first part of our study, we evaluated for the first time that the effects of supplementation with RHE in the tMCAO model. The results of this evaluation revealed that pretreatment with RHE significantly reduced the BBB permeability disruption and cerebral edema, and more importantly, relieved brain infarction and neurological deﬁcit scores after focal cerebral ischemia reperfusion.

Brain inﬂammation has recently emerged as a key player in the pathogenesis of I/R injury (1). It has been shown that RHEs is important for the prevention of phosphorylation of MAPKs, thereby blocking NF-kB activation, which in turn leads to decreased expression of iNOS and Cyclooxygenase-2 (COX-2) (2). Similarly, leukocytes activities, pro-inﬂammatory enzymes and mediators such as NO, IL-1β, and TNF-α were suppressed by REs during inflammation (3).

Oxidative stress is one of the most important mechanisms involved in pathogenesis of ischemic injuries and results in BBB disruption and neuronal death (4). It has also been reported that RHEs decrease lipid peroxidation and hydroxyl anion radical and hydrogen peroxide activities in rat serum, liver, kidney, heart, and brain tissues (5). Based on the mentioned evidences, it can be concluded that RHEs possesse strong antioxidant properties (6), even more than the phenol compounds (7).

**Table 1:** the body weight changes of animals on the 1st and 30^th^day 0, 50, 75, and 100 mg/Kg/day of RHE administration.

	1th day of pretreatment				30th pretreatment			
	0	50	75	100	0	50	75	100
Body weigh	296.6±2.4	289.3±3.4	297.6±4.0	295.9±2.5	331.6±3.1	323.3±3.6	327.5±3.3	315±2.8
Weight gain	0	0	0	0	35±0.7	33.9±0.2	29.8±0.6	1 9 . 1 ± 0 . 3 (p=0.009)

**Table 2:** The distribution of neurologic deﬁcit score in each group

**No. **	**Experimental groups **	**NDS in each groups (N) **						**Total(N) **	**Median**	**Premature death **		**Statistical results **
		0	1	2	3	4	5					
1	Dose 0	1	2	6	8	1	0	18	2.5	2	1:2 sig. (0.000)	
2	Dose 50	7	8	3	0	0	0	18	1	0	1:3 sig. (0.000)	
3	Dose 75	7	9	2	0	0	0	18	1	0	1:4 sig. (0.000)	
4	Dose 100	8	10	0	0	0	0	18	1	1	3:4 nonsig.	
-	Total (N)	23	29	11	8	1	0	72	-	3	2:3, 2:4nonsig.	

**NDS:** neurologic deﬁcit score; **N:** the number of cases in each group; **sig:** signiﬁcant; **nosing:** non-signiﬁcant.

The results showed that the administration of RHE resulted in an effective improvement of cerebral infarction, and neurological scores, also it is able to successfully attenuate BBB permeability which in turn lead to the reduction of brain edema. Thus, RHE protects against BBB disruption induced by acute ischemic injury through reducing brain edema, intracranial pressure, and restoring cerebral blood flow and energy. 

Recent studies have reported that the phenolic compounds from Rosemary (*Rosmarinus officinalis* L.) not only attenuate oxidative stress (8,9) but also reduce cholesterol and triacyl-glycerols levels (10) in serum. It has been suggested that a biological active plasma level can be achieved by oral consumption of the concentration of 5% in the extract (11). Consistent with these studies, the second point of our study exhibited that the RHE supplementation significantly improved the TG/HDL-c and LDL/HDL-c ratios in a dose-dependent manner.

 In conclusion, the present study demonstrated that cerebral ischemic tolerance induced by RHE pretreatment leads to a significant reduction of acute ischemic stroke lesion even in “ischemic penumbra” tissue that is extremely sensitive to hypoxic-ischemic damage. Therefore, RHE could markedly improve stroke outcome. Based on the mentioned evidences, although further works are required to clarify this issue, ischemic tolerance induced by RHE supplementation can be useful as a practical therapeutic in related diseases. 
